# 

**Published:** 1869-06

**Authors:** 


### ARTICLE VIII.

*Tennessee State Dental Association.*

The time and place of the Third Annual Meeting of the Tennessee State Dental Association having been changed from Lookout Mountain, will convene at Nashville on Thursday, July 22, 1868.

Any contributions in the way of original Essays upon Medical or Dental Science will be thankfully received by the Association.

Wm. M. R. Johns, Secretary,
Sommerville.

Wm. T. Arrington, President,

Memphis.

*Charleston S. C. Dental Association.*—We have received from the Secretary a copy of the Constitution of this Association, which was organized in December 1867, with Drs. Patrick, Solomon, Brown, Chupein, Muckenfuss, Bull, Shaffer and White as original members. The regular meetings are held on the second Tuesday evening of each month, and its present officers are Dr. J. B. Patricks, President,—Dr. W. S. Brown, Vice-President,—Dr. T. F. Chupein, Secretary and Treasurer.

*The Meeting of the American Dental Association.*—The attention of those of our readers who purpose attending the next meeting of the American Dental Association, which is to be held at Saratoga on the first Tuesday in August, is directed to the communication of Dr. Ambler, Chairman of Committee of Arrangements, which is published in the present number of the *Journal.* As this meeting is held at a time when Saratoga is thronged with guests, it will be necessary for all who desire good accomodations to notify Dr. Ambler; otherwise it may be impossible to secure rooms at the hotels.

